# Tape Stripping Technique for Stratum Corneum Protein Analysis

**DOI:** 10.1038/srep19918

**Published:** 2016-01-28

**Authors:** Maja-Lisa Clausen, H.-C. Slotved, Karen A. Krogfelt, Tove Agner

**Affiliations:** 1 Department of Dermatology, Bispebjerg Hospital, University of Copenhagen, Copenhagen, Denmark; 2 Department of Microbiology and Infection Control, Statens Serum Institut, Copenhagen, Denmark

## Abstract

The aim of this study was to investigate the amount of protein in stratum corneum in atopic dermatitis (AD) patients and healthy controls, using tape stripping technique. Furthermore, to compare two different methods for protein assessment. Tape stripping was performed in AD patients and healthy controls to collect stratum corneum samples and subsequently analysed with two different methods: Squame Scan, which gives an estimate of total protein (soluble and insoluble) and Micro BCA protein determination kit which measures soluble protein. Significant differences in cumulative protein content between AD lesional, AD non-lesional and healthy control skin was found using the Squame Scan as well as the Micro BCA protein determination kit. AD patients had significantly lower amount of protein, both total protein and soluble protein compared to healthy controls. Furthermore, soluble protein formed 82% of total protein in AD lesional skin, compared to 17–24% for AD non-lesional skin and healthy control. A decreasing amount of total protein with increasing stratum corneum depth was found for all skin types. Significant differences in stratum corneum protein content between AD lesional, AD non-lesional and healthy control skin were revealed, independent of method used.

Atopic dermatitis (AD) is an inflammatory skin disease with increasing prevalence worldwide^1^. Although generally considered primarily an immune disease, AD is greatly linked to skin barrier dysfunction such as filaggrin mutations, changed lipid composition and altered antimicrobial response^2^. Despite these findings, the pathogenesis is still unclear, and skin barrier research, with focus on stratum corneum, is fundamental in creating a better understanding of this disease.

Skin biopsies are frequently used for studies on immunological parameters as well as skin barrier function. However, minimal invasive methods are desirable to enable more dynamic and repetitive skin sampling over time in relation to treatment, environment and disease flares. Furthermore, it is essential to reduce discomfort, potential infection and scarring caused by skin biopsies. Investigating the skin barrier requires specific methods for obtaining solely the stratum corneum, and minimizing inferring substances from deeper layers of the epidermis, and therefore tape stripping has become an increasingly popular method and proven valuable for stratum corneum research^3^,^4^,^5^. Tape stripping has been used for measurement of AMPs, lipids, NMF, aquaporins and many other players in the stratum corneum^4^,^6^,^7^,^8^, and assessment of protein content is an essential baseline for such studies.

Different methods have been used over time to evaluate protein content of tape strips including colorimetrical methods which evaluate protein content directly on the tape^9^,^10^,^11^, as well as different extraction procedures^3^,^12^,^13^,^14^ to retrieve the proteins from the tape.

The overall aim of this study was to compare protein content in stratum corneum samples from AD skin (lesional and non-lesional) and healthy control (HC) skin, using tape-stripping technique. A second aim was to compare two different methods for protein assessment. Additionally different variables such as pressure time, sonication time and lysis buffer, all important for tape stripping technique, are evaluated.

## Results

### Non-invasive measurements

Non-invasive measurements of TEWL and skin pH were performed on all AD patients and HC1 (Table 1). SCORAD was performed on all AD patients (Table 1). There was no significant difference in skin pH between AD patients and HC. A significantly higher TEWL was found for AD non-lesional skin compared to HC skin (p = 0.04) (Table 1).

### Protein extraction procedure - different variables

#### Effect of pressure and sonication time

**Figure 1:**
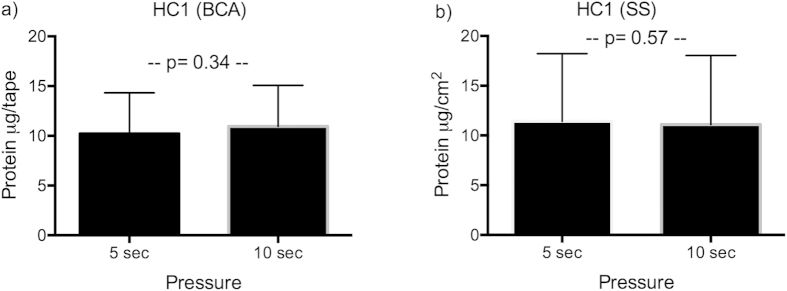
Effect of pressure time on protein yield. Protein amount in tape strips from HC1 receiving either 5 sec or 10 sec of standardised pressure determined by BCA (**a**) and Squame Scan (**b**). There was no significant difference in protein yield between 5 or 10 sec. AD: atopic dermatitis; HC: healthy control; SS: Squame Scan, BCA: Micro BCA protein determination kit.

All HC1 had 2 × 35 tapes collected using pressure of 5 sec and 10 sec respectively (Table 2). There was no significant difference in protein yield between the two pressure times, determined by both BCA (p = 0.34) and Squame Scan (p = 0.57) (Fig. 1).

**Figure 2:**
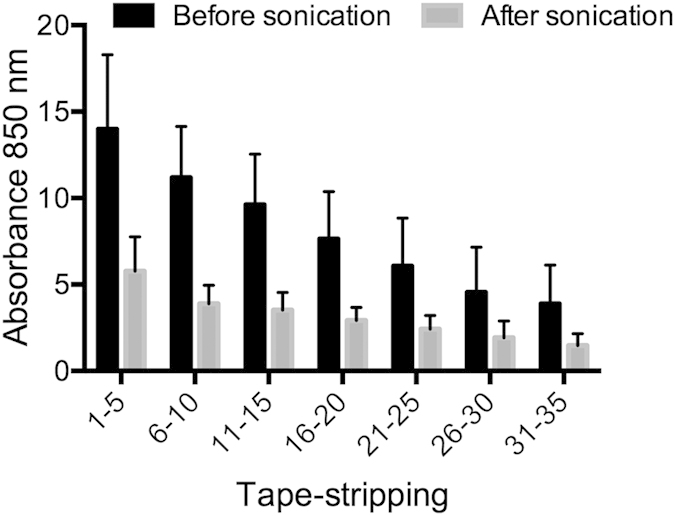
OD measurements by Squame Scan (AD and HC1), before and after sonication, revealing a significant reduction in OD for all depths of stratum corneum. P < 0.0001 for all levels. OD: optical density. Number of samples = 161.

**Figure 3:**
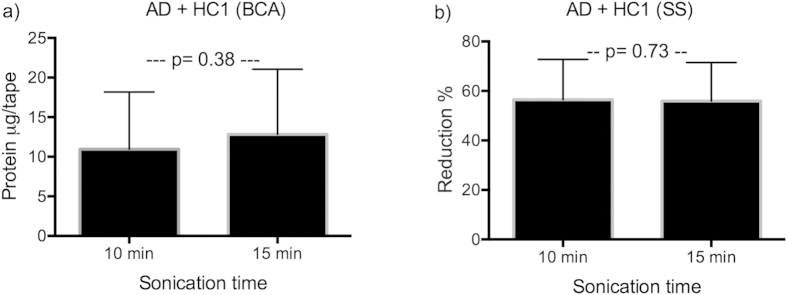
Effect of sonication time on protein yield. (**a**) Protein determination by BCA in tapes from AD and HC1 receiving either 10 or 15 min of sonication. No significant difference was found in protein yield. (**b**) Reduction in Squame Scan OD after sonication of either 10 or 15 minutes in tapes from AD and HC1. There was no significant difference in protein yield. AD: atopic dermatitis; HC: healthy control; SS: Squame Scan, BCA: Micro BCA protein determination kit.

To evaluate the effect of sonication on yield of protein extracted from the tapes, tapes were scanned by Squame Scan before and after sonication, to measure changes in absorbance. OD measurements decreased significantly (p < 0.001) between 50–90% after sonication (Fig. 2). 10 tapes were collected on all AD patients and HC1 (Table 1), which received only 10 min of sonication. There was no difference in retrieved amount of protein between 10 min or 15 min of sonication, determined by both BCA (p = 0.38) and Squame Scan (p = 0.73) (Fig. 3).

#### Protease inhibitor and extraction buffer

The use of protease inhibitor did not increase protein yield compared to standard (PBS + ultrasound) (S1). The use of T-PER buffer (protein extraction buffer) all resulted in identical OD in the BCA kit, therefore no difference could be determined in the samples (S1). The T-PER buffer was tested with spike standards prepared using the provided albumin in the BCA kit. All standards prepared with T-PER gave identical OD (OD = −0.01), apart from 40 μg/ml (OD = 0.01) and 200 μg/ml (OD = 0.5) (S1).

Control samples with PBS (Table 2) all gave OD measurements similar to blank, indicating no interfering substances (S1). However, all samples containing T-PER (Table 2) gave similar higher OD measurement, equal to a protein content of around 6–10 μg/ml in all samples, suggesting that T-PER interferes with the protein analysis (S1).

### Protein measurements

#### Protein content in AD vs. HC

Cumulative soluble protein content determined with BCA showed significantly lower protein for AD lesional skin and non-lesional skin than HC (p = 0.0006 and p < 0.0001) but no significant differences between AD lesional skin and non-lesional skin (p = 0.9)(Table 3). Cumulative total protein content determined by Squame Scan also showed significantly lower protein for AD lesional skin and non-lesional skin than for HC (p < 0.0001 and p = 0.0042), and significantly lower for AD lesional skin than for AD non-lesional skin (p < 0.0001) (Table 3). Calculated as μg/cm^2^, the proportion of soluble protein (BCA) out of total protein (Squame Scan) for AD non-lesional skin and HC formed 16.9% and 24.2% respectively. However, for AD lesional skin, soluble protein (BCA) formed 81.6% of total protein (Squame Scan) (Table 3). Great inter-individual differences measured as coefficients of variance (CV%) were found both for BCA (22–56%) and Squame Scan (48–66%) and increased with stratum corneum depth (Tables 3, S2).

#### Protein content in the depth of stratum corneum

**Figure 4:**
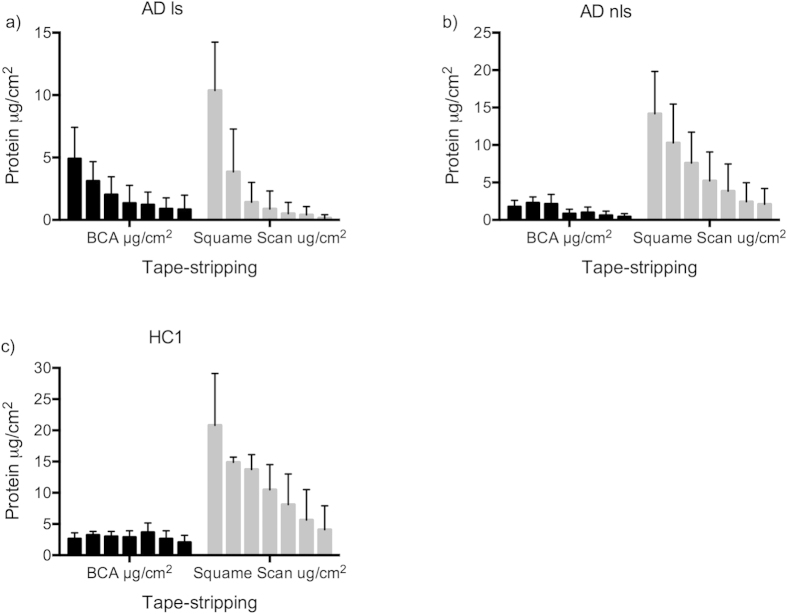
Comparison of protein amount in tape strips, determined by BCA μg/cm^2^, and calculated by Squame Scan μg/cm^2^ (OD = 0.623x + 2.703). Mean protein content and standard deviation. (**a**) AD lesional skin, (**b**) AD non-lesional skin, (**c**) Healthy control 1.

BCA determination showed a distinct profile for each skin type (Fig. 4). For HC skin, a steady level of soluble protein was found through the depth of stratum corneum, whereas for AD non-lesional skin, a significant decrease in soluble protein content was observed after tape no. 15 (p = 0.015). AD lesional skin showed decreasing levels of soluble protein with increasing stratum corneum depth (Fig. 4). Squame Scan measurements showed a clear and significant decrease of protein content throughout the depth of stratum corneum for both HC and AD lesional and non-lesional skin, with a reduction of 7–63% in amount of protein for each set of 5 tapes (S2).

### Correlation of methods for protein measurements

**Figure 5:**
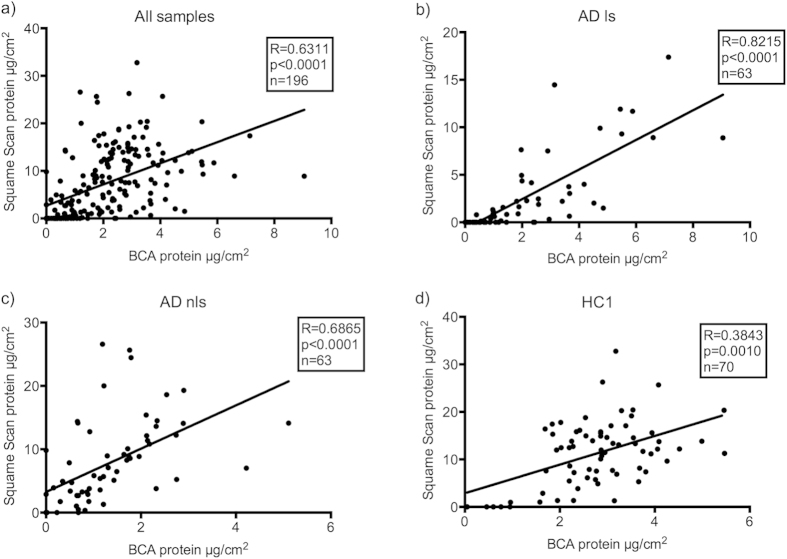
Linear regression between protein determinations made by BCA or calculated by Squame Scan (OD = 0.623x + 2.703). Protein is measured as μg/cm^2^. Significant positive correlation between the two methods was found for all samples. (**a**) All samples, AD + HC; (b) AD lesional skin, (**c**) AD non-lesional skin, (**d**) Healthy control 1. SS: Squame Scan, n: number of samples, AD: atopic dermatitis, HC: healthy control.

Protein quantification was performed both by calculation of total protein using Squame Scan and by measuring soluble protein using BCA. The overall Spearman correlation for all samples (AD and HC, n = 196) between BCA and Squame Scan was R = 0.63 (CI: 0.54–71; p < 0.0001). Correlation was higher when comparing only AD lesional skin (R = 0.82, CI: 0.72–0.89) and AD non-lesional skin (R = 0.69 CI: 0.52–0.80), but lower for HC skin (R = 0.38, CO: 0.15–0.57) (Fig. 5).

## Discussion

Comparing the skin of AD patients and HC, we found protein content to be significantly lower in AD skin than HC, independent of method used for assessment. This is in agreement with a previous report^15^ and is most likely related to differences in barrier properties, as indicated by significant differences in TEWL (Table 1) and a thinner stratum corneum of AD patients, most pronounced in lesional skin^15^. Anatomical site has shown to be of importance when performing tape stripping^16^, and therefore all samples were collected from the volar forearm. In HC and AD non-lesional skin, soluble protein was found to compose 17–24% of total proteins, whereas in AD lesional skin, soluble protein composed 82% of total protein, indicating a changed stratum corneum in lesional AD skin. Since 85% of total protein is believed to originate from insoluble corneocytes^14^, this finding indicates the presence of increased amount of intercellular matrix relative to corneocytes in lesional AD skin. In the present study patients with severe AD were included as can be seen from SCORAD values (Table 1), and significant scaling and inflammation were typically found in lesional skin, both which could be assumed to lead to this relative increase in intercellular matrix and fewer corneocytes. Filaggrin mutations may also diminish the intercellular adherence and lead to shedding of corneocytes and thereby thinner stratum corneum, which would explain the significant difference in the proportion of soluble protein relative to total protein observed for AD lesional skin.

The level of total protein, measured by Squame Scan, showed a progressive decrease with stratum corneum depth for all skin samples, in line with previous reports^9^,^17^. This is believed to be due to increasing corneocyte cohesion in the deeper layers of stratum corneum, leading to fewer corneocytes collected on the tape strip^18^,^19^. The amount of soluble protein, showed a stable level throughout the stratum corneum for HC, but for AD non-lesional skin a stable level restricted to the upper layers, and a decrease below tape no 15 and AD lesional skin showed decreasing levels of soluble protein all through stratum corneum. Soluble protein mainly originates from the intercellular matrix, and the constant level found in healthy skin throughout stratum corneum indicates a stable structure of the stratum corneum, while the decreased values in AD indicates less amount of intercellular matrix, in particular in inflamed skin in lower layers^18^.

Both methods revealed great inter-individual variances (CV%) in protein content of stratum corneum (Table 3). Since technique and procedure for tape-stripping and protein extraction were standardised in this study, individual stratum corneum properties are believed to explain this inhomogeneity.

With respect to the two different methods for protein determination: Squame Scan and BCA, an overall statistically significant positive correlation between the two methods was found. However, since the two methods essentially measure different components, Squame Scan measures total protein, soluble and insoluble, whereas BCA measures only soluble protein, it is not surprising that the correlation is not higher. Since the amount of soluble protein in AD skin decreases through stratum corneum, compared to a steady level in HC, this explains the higher correlation between the methods in AD skin. In a previous study a high correlation was reported between Squame Scan and protein extracted from D-squame tape (R = 0.85)^11^. This difference in correlation is probably explained by differences in protocol, with respect to buffers in particular, as well as temperature. In the present study, PBS buffer and temperature 4 °C was used to extract soluble protein, while previously NaOH and 37 °C was used for the purpose of extracting total protein, which would lead to a better correlation with the Squame Scan method.

In our study, the use of protease inhibitor, increased pressure time or sonication time did not influence soluble protein yield, indicating that PBS buffer and the described protocol was sufficient to extract soluble proteins from the tape. We found that the proportion of soluble protein out of total protein accounted for 17–24% in AD non lesional skin and HC skin, which agrees with previous reports where the use of PBS, tween and sonication was used to extract soluble protein and compared to total protein extracted by the use of methanol, NaOH and HCl^14^.

The use of Squame Scan is a quick and easy method to evaluate protein content of tape strips (D-squame), and a practical method for measuring total protein. However, the scan covers 1.8 cm^2^, which corresponds to 47% of the tape strip (D-squame). Uneven tear and stripping, which is difficult to avoid, might negatively influence results and correlation between Squame Scan and BCA. The uneven protein collection on tape strips has previously been reported^20^ and it is therefore recommended to use the entire tape for more precise protein determination. As compared to the limited tape area investigated by the Squame Scan, the BCA method measures protein from the total tape area.

Many different tapes have been used in previous studies using tape stripping. In previous reports no significant differences in cumulative protein removal between different types of tapes^14^,^17^ have been reported. In the present study, we used D-squame, which has been commonly used for this purpose^5^,^21^,^22^,^23^.

Based on results from our study, we recommend a protocol of a total of 1–15 tapes, standardised pressure of 5 sec., 1 mL of PBS buffer for each tape, and sonication time of 10 minutes in cooled ultrasonic bath (Table 4). Depending on the expected depth in stratum corneum of the compound of interest, one might consider tape stripping to deeper levels. The use of PBS buffer was in the present study sufficient to extract soluble proteins, however, the addition of tween to PBS is previously reported with good results^3^,^14^. Avoidance of additional substances will minimize interference with later ELISA analysis. The use of protease inhibitor did not increase protein yield, and the lysis buffer T-PER interfered with the micro BCA Kit, and therefore cannot be recommended. Other groups have used NaOH buffer for protein extraction with good results^17^,^20^, and neutralised with HCl. However consideration should be made that strong buffers might interfere with ELISA analysis or harm fragile proteins, depending on compound of interest.

To gather the most information on soluble and insoluble protein, ideally both methods should be applied when analysing proteins in the stratum corneum. However, depending on the study and information needed, using the BCA analysis would in most cases be sufficient to provide a baseline for analysing other soluble proteins and compounds like inflammatory markers.

## Conclusions

Focus in recent years on a better understanding of the skin barrier has highlighted the importance of skin barrier research. Tape stripping has become an important method for analysing parameters in the stratum corneum, and have been used for quantification of AMPs, lipids, interleukins, NMF and more^4^,^6^,^8^,^24^,^25^. When measuring components like these, a baseline protein level is essential for comparison and quantification. Tape stripping has great advantages in being cheap, easy, and quick, it does not influence inflammatory components, and it is painless and leaves no scar. Tape stripping is limited to studies in the epidermis, predominantly the stratum corneum, and therefore cannot be used for investigating the dermis. So far, tape stripping has been used for quantifying numerous compounds in the stratum corneum, however, whether tape stripping can also be used in a broader analysis of the skin, e.g. epidermal cells and histological analyses, might be determined in future studies.

In this study we report significant differences in stratum corneum integrity/protein content between AD skin and HC, using the Squame Scan as well as the Micro BCA protein determination kit, and confirm a significant positive correlation between the two methods. Additionally we propose a standardised method for the extraction of soluble protein, with minimal handling and using a minimum of interfering substances.

## Methods and Materials

### Study material

AD patients (n = 9), 6 women and 3 men, age 19–67, were included in the study. All patients fulfilled inclusion criteria: > 18 year, AD according to UK criteria^26^. Skin was left untreated for at least 1 week before tape strip collection. Two groups of healthy controls without any history or manifestations of AD or other skin diseases were included: Healthy controls (HC group 1) n = 5, 2 women and 3 men, age 21–59, and healthy controls (HC group 2) n = 4, 3 women and 1 man, age 30–59. HC1 had tape strips collected for protein analysis, and HC2 had tape strips collected for supplementary control samples. Stratum corneum tape strips and non-invasive measurements of Trans epidermal Water Loss (TEWL) and skin pH were collected for all AD patients and HC1. For AD patients, disease severity was assessed using Scoring Atopic Dermatitis (SCORAD)^27^. The National Committee in Health Research Ethics, Copenhagen, Denmark, approved the study (H-1-2014-039), and the methods were carried out in accordance with this approval. All participants provided informed written consent.

### Non-invasive measurements and scores

Non-invasive measurements of TEWL and skin pH were performed under standardized conditions. Before measurements, patients were given time to adapt to room conditions and the same investigator (MLC) performed all measurements. TEWL was measured on non-lesional skin, on the volar side of the distal forearm, using the Dermlab open chamber Evaporimeter (Cortex Technology, Hadsund, Denmark), according to guidelines^28^. Skin pH was measured on non-lesional skin, on the volar side of the distal forearm, using a skin-pH-meter (©Mettler-Toledo, Greisensee, Switzerland)^29^. All measurements of TEWL and skin pH were performed in triplicates and the registered values indicate the mean of the three measurements. Disease severity was determined by SCORAD (score range 0–103)^30^. Scoring is based on extent of eczema together with six clinical features: erythema/darkening, oedema/population, oozing/crust, excoriations, lichenification and dryness as well as two subjective symptoms: prurigo and sleep quality^27^.

### Tape Stripping - for protein analysis

Stratum corneum was collected by tape stripping using D-squame (D-squame®, CuDerm, Dallas TX, USA). 35 consecutive tape strips were collected from the same area, and pooled in groups of 5: 1–5, 6–10, 11–15, 16–20, 21–25, 26–30, 31–35 for protein determination (Table 2).

#### AD patients

All AD patients had 35 consecutive tapes collected from lesional skin and 35 consecutive tapes from non-lesional skin. The first tape was discarded to eliminate dirt and remnants of skin products. Each tape was pressed against the skin with standardized pressure for 10 seconds (sec) using a standardized pressurizer (D500–D-squame® pressure instrument, CuDerm, Dallas, TX, USA) and placed in tubes on ice. Non-lesional skin samples were collected from the volar side of the forearm and lesional skin samples were collected from the arm at location of eczema.

#### HC

HC1 (n = 5) had 35 consecutive tape strips collected from the volar side of the forearm receiving 10 sec of standardized pressure. HC2 (n = 4) had 4 × 10 tape strips, receiving 10 sec of pressure, collected from the volar side of the forearm to test if protease inhibitor or protein extraction buffer would increase the yield of protein extraction (Table 1). All samples received 15 min of sonication in iced water (0–4 °C) in an ultrasonic bath (Bransonic® 5510, Branson Ultrasonics, Danbury, CT, USA) (Table 2).

All tapes were scanned for density using Squame Scan 850 (D501 D-squame Scan 850, CuDerm, Dallas Tx, USA)^11^, before and after sonication. All tapes were handled wearing gloves at all times to avoid protein contamination.

### Protein extraction procedure - different variables

#### Sonication time and pressure

For AD (n = 9) patients and HC1 (n = 5), additional 10 tape strips were collected with 10 sec of pressure, which received only 10 min of sonication (Table 2). The 10 tapes were collected from lesional skin of AD patients. For HC1 (n = 5) an additional 35 tape strips were collected with standardized pressure of only 5 sec, and 15 min of sonication (Table 2). All tape strips were collected from the arms. All tapes were scanned for density using Squame Scan 850^11^, before and after sonication. All tapes were handled wearing gloves at all times to avoid protein contamination.

#### Protease inhibitor and extraction buffer

HC2 (n = 4) had 4 × 10 tape strips collected from skin of the forearm with applied pressure of 10 sec (Table 2). Each set of 10 tapes was treated with either: 1) PBS and 15 min sonication, 2) PBS and protease inhibitor (cOmplete, Roche Diagnostics Cor., Indianapolis, USA) and 15 min sonication, 3) T-PER buffer (Tissue protein extraction reagent, Thermo Fischer Scientific Inc., Waltham, MA USA), or 4) T-PER buffer and 15 min sonication. To test for influencing substances in the tape, PBS, protease inhibitor or TER buffer, supplementary controls were prepared from: 1) PBS + 15 min sonication, 2) clean tape + PBS + 15 min sonication, 3) clean tape + PBS + protease inhibitor + 15 min sonication, 4) TER buffer, 5) clean tape + TER buffer, 6) clean tape + TER buffer + 15 min sonication (Table 1).

### Determination of protein content in stratum corneum samples

#### BCA - protein determination kit

PBS buffer (1000 μL) was added to each individual tape in tubes, followed by 15 min of sonication, in iced water (0–4 °C) in an ultrasonic bath (Bransonic® 5510). Samples were pooled for each set of 5 tapes (1–5, 6–10, 11–15, 16–20, 21–25, 26–30, 31–35), and stored at −80 °C until further analysis. Micro BCA^TM^ Protein Assay Kit (Thermo Fisher Scientific Inc., Waltham, MA USA) was performed according to protocol, to determine soluble protein extracted in the PBS buffer. In brief, standards were prepared in PBS buffer (pH 7.4) and 150 μl of standards and samples were added to micro plates in triplicates. Working reagent solution made from Reagent MA, Reagent MB and Reagent MC (25:24:1) was added to each well, solutions were mixed and the plates were incubated for 2 hours at 37 °C. The plates were left to cool until room temperature, and read using 570 nm absorbance on a micro plate reader (Thermo Fischer Scientific). Protein determination by BCA is calculated both as μg/tape and μg/cm^2^.

#### Squame Scan

All tapes were scanned before and after the addition of PBS buffer and sonication, using Squame Scan 850 according to protocol. In brief, the instrument is calibrated with the applied glass, and empty D-squame tape strip is used for blank reference for each set of readings. Protein content is determined using the standard formula OD = 0.623x + 2.703^11^.

### Statistics

Statistical analysis was carried out using Prism 6 GraphPad, Mann-Whitney non-parametric test, unpaired t-test, linear regression analysis and spearman correlation coefficient. All negative OD measurements are interpreted as having no protein, and value was set as 0.

## Additional Information

**How to cite this article**: Clausen, M.-L. *et al.* Tape Stripping Technique for Stratum Corneum Protein Analysis. *Sci. Rep.* **6**, 19918; doi: 10.1038/srep19918 (2016).

## Supplementary Material

Supplementary Dataset 1

## Figures and Tables

**Table 1:** Non-invasive measurements, AD patients (n = 9) and HC1 (n = 5).

	AD nls median *(range)*	HC median *(range)*	p-value
Skin pH	5.6 *(4.4-8.49)*	5.3 *(4.9-6.5)*	0.9
TEWL	9.2 *(4.6-16.8)*	5.9 *(4.9 - 8.2)*	0.04
SCORAD	36.8 *(7.7-61.5)*	—	—

Non-invasive measurements of AD patients and HC1. Median value and range of skin pH, TEWL and SCORAD. Significantly higher TEWL was found for AD non-lesional skin compared to HC skin.

AD: atopic dermatitis; HC: healthy control; nls: non-lesional skin; TEWL: trans epidermal water loss; SCORAD: Scoring atopic dermatitis.

**Table 2:** Collected tape strips.

	Number of participants	Number of tape strips	Pressure time	Sonication time	No of pooled samples per participant
AD samples	9	35 ls	10 sec	15 min	7
		35 nls	10 sec	15 min	7
		10 nls	10 sec	10 min	2
HC1 samples	5	35 nls	10 sec	15 min	7
		35 nls	5 sec	15 min	7
		10 nls	10 sec	10 min	2
HC2 samples	4	10 nls (PBS)	10 sec	15 min	2
		10 nls (PBS + PI)	10 sec	15 min	2
		10 nls (T-PER)	10 sec	no sonication	2
		10 nls (T-PER)	10 sec	15 min	2
Control samples	6	1) PBS + 15 min sonication 2) Clean tape + PBS + 15 min sonication 3) Clean tape + PBS + protease inhibitor + 15 min sonication 4) T-PER buffer 5) Clean tape + T-PER buffer 6) Clean tape + T-PER buffer + 15 min sonication			

Overview of collected tape strips from AD patients and HC. All samples were pooled for each set of 5 tape strips. AD: atopic dermatitis; HC: healthy control, ls: lesional skin, nls: non-lesional skin. PI: protease inhibitor.

**Table 3:** Cumulative protein content in tape strips.

	AD ls (n = 63)	AD nls (n = 63)	HC1 (n = 35)	Significant difference of protein content between groups (p-value)
BCA μg/tape ± SD *(CV%)*	55 ± 31 *(56.2%)*	34 ± 9 *(25.5%)*	77 ± 17 *(22.8%)*	HC vs. AD ls: Yes (p = 0.0006)
				HC vs. AD nls: Yes (p < 0.0001)
				AD nls vs. AD ls: No (p = 0.0926)
Squame Scan μg/cm^2^ ± SD *(CV%)*	18 ± 11 *(66.6%)*	54 ± 21 *(48.4%)*	78 ± 35 *(51.7%)*	HC vs. AD ls: Yes (p < 0.0001)
				HC vs. AD nls: Yes (p = 0.0042)
				AD nls vs. AD ls: Yes (p < 0.0001)
Soluble protein (BCA) in percentage of total protein (SS)	82%	17%	24%	Measured as μg/cm^2^

Cumulative protein content in tape strips of stratum corneum from AD patients and HC. Protein content determined by BCA and Squame Scan. Significantly lower amount of protein in AD compared to HC determined by both BCA and Squame Scan. AD: atopic dermatitis, HC: healthy controls, ls: lesional skin, nls: non-lesional skin, SD: standard deviation. n: number of samples.

**Table 4:** Recommended method for protein extraction from tape strips (D-squame).

	Recommendation	Comment
Number of tapes	*1*–*15*	*After tape no. 15, the amount of soluble protein decreased for AD. Number of tapes should reflect the expected depth of the compound of interest.*
Buffer	*PBS*	*Adding protease-inhibitor or using lysis buffer did not improve protein extraction.*
		*PBS buffer extracts soluble proteins, however, to extract all proteins (insoluble* + *soluble) a different buffer should be considered.*
Standardised pressure	*5* *sec*	*No significant difference in protein yield between 5 and 10* sec *of pressure.*
Sonication time	*10* min *in cooled sonication bath*	*No significant difference in protein yield between 10 and 15* min*utes of sonication.*
