# Supplementary material for: Tape Stripping Technique for Stratum Corneum Protein Analysis
Source: Sci Rep. 2016 Jan 28;6:19918. doi: 10.1038/srep19918 (PMC4730153; doi:10.1038/srep19918)
Supplement: Supplementary Dataset 1 [file srep19918-s1.doc]

Tape Stripping Technique for Stratum Corneum Protein Analysis

Maja-Lisa Clausen^1^, H-C Slotved^2^, Karen A. Krogfelt^2^, Tove Agner^1^

1) Department of Dermatology, Bispebjerg Hospital, University of Copenhagen, Copenhagen, Denmark.

2) Department of Microbiology and Infection Control, Statens Serum Institut, Copenhagen, Denmark.

Supplementary material:

Figure S1:

Figure S1: supplementary controls. a) Protein μg/tape in tape strips from HC2 (n=4), tested for different variables. Protein content determined by BCA. b) Protein determination in supplementary control samples.

UL: sonication, PI: protease inhibitor, T-PER: protein extraction/lysis buffer

Table S2:

| AD non-lesional | | | | | |
| --- | --- | --- | --- | --- | --- |
| Tape | BCA μg/tape ±SD *(CV%)* | BCA μg/cm^2^ | SS μg/cm^2^ ±SD *(CV%)* | Reduction in % (SS) | BCA in % of SS |
|
| 1-5 | 6.68 ± 3.29 *(49.3%)* | 1.76 | 16.64 ± 6.01 *(46.5%)* |  | 10.6% |
| 6-10 | 8.67 ± 3.01 *(34.7%)* | 2.28 | 12.93 ± 5.85 *(53.1%)* | 22.3% | 17.7% |
| 11-15 | 8.10 ± 4.87 *(60.2%)* | 2.13 | 9.64 ± 5.10 *(59.0%)* | 25.4% | 22.1% |
| 16-20 | 3.22 ± 2.24 *(69.6%)* | 0.85 | 6.48 ± 4.09 *(67.3%)* | 32.8% | 13.1% |
| 21-25 | 3.74 ± 2.82 *(75.3%)* | 0.98 | 4.11 ± 3.30 *(82.1%)* | 36.6% | 24.0% |
| 26-30 | 2.36 ± 2.12 *(89.8%)* | 0.62 | 2.13 ± 2.29 *(106.1%)* | 48.2% | 29.2% |
| 31-35 | 1.62 ± 1.61 *(98.9%)* | 0.43 | 1.80 ± 1.73 *(95.9%)* | 15.6% | 23.8% |
| *Cumulative* | *34.39 ± 8.77 (25.5%)* | *9.05* | *53.72 ± 20.9 (48.4%)* |  | *16.9%* |
| AD lesional | | | | | |
|  | BCA μg/tape ±SD *(CV%)* | BCA μg/cm^2^ | SS μg/cm^2^ ±SD *(CV%)* | Reduction in % (SS) | BCA in % of SS |
| 1-5 | 18.63 ± 9.56 *(51.3%)* | 4.90 | 10.37 ± 3.87 *(47.3%)* |  | 47.3% |
| 6-10 | 11.85 ± 5.87 *(49.5%)* | 3.12 | 3.85 ± 3.43 *(89.6%)* | 62.8% | 80.9% |
| 11-15 | 7.69 ± 5.42 (70.5%) | 2.02 | 1.41 ± 1.59 *(110.7%)* | 63.4% | 143.3% |
| 16-20 | 5.10 ± 5.41 *(106.0%)* | 1.34 | 0.88 ± 1.43 *(156.6%)* | 37.6% | 152.2% |
| 21-25 | 4.61 ± 3.86 *(83.7%)* | 1.21 | 0.50 ± 0.91 *(172.4%)* | 42.8% | 240.5% |
| 26-30 | 3.41 ± 3.33 *(97.7%)* | 0.90 | 0.40 ± 0.67 *(160.8%)* | 20.4% | 223.5% |
| 31-35 | 3.22 ± 4.30 *(133.6%)* | 0.85 | 0.16 ± 0.25 *(150.7%)* | 59.7% | 523.3% |
| *Cumulative* | *54.51 ± 30.59 (56.1%)* | *14.35* | *17.59 ± 10.93 (66.6%)* |  | *81.6%* |
| HC1 | | | | | |
|  | BCA μg/tape ±SD *(CV%)* | BCA μg/cm^2^ | SS μg/cm^2^ ±SD *(CV%)* | Reduction in % (SS) | BCA in % of SS |
|
| 1-5 | 10.00 ± 3.68 *(36.8%)* | 2.73 | 20.80 ± 11.28 *(58.7%)* |  | 13.1% |
| 6-10 | 12.32 ± 2.12 *(17.2%)* | 3.32 | 14.90 ± 6.13 *(48.1%)* | 28.4% | 22.3% |
| 11-15 | 11.46 ± 3.02 *(26.4%)* | 3.39 | 13.75 ± 6.0 *(45.5%)* | 7.7% | 24.7% |
| 16-20 | 11.01 ± 3.86 *(35.1%)* | 2.72 | 10.49 ± 5.59 *(57.9%)* | 23.7% | 25.9% |
| 21-25 | 13.90 ± 5.75 *(41.4%)* | 2.99 | 8.10 ± 5.50 *(88.9%)* | 22.8% | 36.9% |
| 26-30 | 10.06 ± 4.84 *(48.1%)* | 2.03 | 5.63 ±4.93 *(83.5%)* | 30.5% | 36.1% |
| 31-35 | 7.81 ± 4.26 *(54.5%)* | 1.63 | 4.10 ± 3.80 *(84.9%)* | 27.2% | 39.7% |
| *Cumulative* | *76.57 ± 17.48 (22.8%)* | *18.82* | *77.78 ± 35.29 (51.7%)* |  | *24.2%* |

Table S2: Protein content in tape strips for each set of pooled tape. Protein content determined by BCA (μg/tape in column 2 and μg/cm^2^ in column 3) and Squame Scan (μg/cm^2^ in column 4). The reduction in protein content for each set of pooled tape, determined by Squame Scan is calculated in column 5. The percentage of soluble protein (BCA) out of total protein (Squame Scan) is stated in column 6.

AD: Atopic dermatitis; HC: healthy controls; SS: Squame Scan.
